# Type and etiology of pediatric epilepsy in Jordan: a multi-center study

**DOI:** 10.17712/nsj.2017.4.20170164

**Published:** 2017-10

**Authors:** Abdelkarim A. Al-Qudah, Abla Albsoul-Younes, Amira T. Masri, Samah K. AbuRahmah, Ibrahim A. Alabadi, Omar A. Nafi, Lubna F. Gharaibeh, Amer A. Murtaja, Lina H. Al-Sakran, Haya A. Arabiat, Abdallah A. Al-Shorman

**Affiliations:** *From the Department of Pediatrics (Al-Qudah, Masri), Faculty of Medicine, Department of Bio pharmaceutics and Clinical Pharmacy (Albsoul-Younes, Alabadi, Gharaibeh, Murtaja, Al-Sakran), Faculty of Pharmacy, Hamdi Mango Centre for Scientific Research (Albsoul-Younes), University of Jordan, Amman, from the Department of Pediatrics (AbuRahmah), Faculty of Medicine, Jordan University of Science and Technology, Irbid, from the Faculty of Medicine (Nafi), University of Mutah, Karak, Jordan, from Salt Hospital (Arabiat), Salt, and from Prince Rahma Hospital (Al-Shorman), Irbid, Ministry of Health, Jordan*

## Abstract

**Objective:**

To study types and etiologies of epilepsy in Jordanian pediatric epileptic patients maintained on antiepileptic drugs using customized classification scheme of International League Against Epilepsy (ILAE) (2010) report.

**Methods:**

This is a cross-sectional, multi-centre study on paediatric epileptic patients on antiepileptic drugs, who were managed in the pediatric neurology clinics at 6 teaching public hospitals in Jordan.

**Results:**

Out Of the 663 patients included in the study, (90.2%) had one seizure type, (53%) of this type were focal seizures followed by generalized seizures (41.5%) and spasms (5.5%). Distinctive constellations were found in 11/663 (1.7%) patients. Benign epilepsies with centrotemporal spikes were the most common electro clinical syndromes 60/221 (27.1%). Epilepsies attributed to structural-metabolic causes were documented in 278/663 (41.9%) patients, unknown causes 268/663(40.4%) and genetic causes in 117/663(17.7%). Most common causes of structural-metabolic group were due to perinatal insults (32%) and most common causes of the genetic group were the presumed genetic electro clinical syndromes (93.1%).

**Conclusion:**

Our study is on pediatric epilepsy, using customized classification scheme from the ILAE 2010 report which showed interesting results about type and etiology of epileptic seizures from developing country with potential impact on the international level.

Classification plays an important role in management of epileptic patients, education and epilepsy research. Since the first classification in 1960, tremendous developments have happened in the fields of neuroimaging, neurophysiology, genomic technology and molecular biology, which paved the road for the report of International League Against Epilepsy (ILAE) commission on classification and terminology of epileptic seizures (2005-2009).^1^ The report has attracted a lot of debate regarding its value compared to the ones previously used and current and future research will test the usefulness and validity of its contents.^2^-^7^ Operational classification of seizure types by the ILAE: Position Paper of the ILAE Commission for Classification and Terminology has been recently published.^2^ Few clinical studies have shown some usefulness of the revised classification of the ILAE report 2010.^8^-^11^ The aim of our study was to determine the classification of mode of onset of seizures, types of epilepsies and their etiology, using the proposed Report of the ILAE Commission on Classification and Terminology of epileptic seizures (2010).

## Methods

This is a cross-sectional, multi-center study, on Jordanian pediatric epileptic patients maintained on antiepileptic drugs, using the proposed report of the ILAE Commission on Classification and Terminology of epileptic seizures (2010).

### Study design

The study was a cross-sectional, multi-center study, where all patients diagnosed as epileptic and maintained on antiepileptic drugs were included consecutively in the study during the study period from September 2013 to January 2016. Age at onset of seizures was from birth to 18 years. The study was carried out in 6 public teaching hospitals. Two of the hospitals were university hospitals, 4 were ministry of health (MOH) hospitals, 3 of the MOH hospitals were affiliated to the university hospitals. Two hospitals were located in the north of Jordan, 3 in the middle and one in the south. Great collaboration is present among these hospitals in different services including clinical, educational and investigations services. Also, when necessary, some unavailable genetic and metabolic investigations were carried out in other medical centers in Jordan or abroad. Clinical data were documented in the study forms directly from the patients, their families and from their clinical records during the study period by the pediatric neurologist working in each hospital.

Classification was carried out by one pediatric neurologist. However; all data in the study forms were reviewed by the first author. Whenever there were questions concerning the classification or etiology or both, the cases were discussed with the assigned pediatric neurologist till the final decision was reached. In one of the MOH hospitals, all patients included in the study were evaluated directly by the first author and the assigned pediatric neurologist (co-author) in that hospital. In another MOH hospital, the first author reviewed all patients’ medical records with the assigned pediatric neurologist (co-author) in that hospital.

### Inclusion and exclusion criteria

All patients aged 2 months-18 years who had a final diagnosis of epilepsy and maintained on antiepileptic drugs were included in the study.^12^ All patients with febrile seizures, acute symptomatic seizures and isolated neonatal seizures were excluded as carried out by Syvertsen et al.^13^

### Ethical approval

The ethical approval for the study conduction was obtained from IRB committee in the hospitals where the study was carried out. Guardian, of eligible patient was asked for permission of enrolment of his child. After obtaining consent from the child’s legal guardians, a case report form was used to collect pertinent information.

### Statistics

The data were analyzed using the IBM^®^ SPSS^®^ Statistics for Windows version 21.0 (IBMCorp, Armonk, NY, USA). Categorical data were summarized using frequencies and percentages n (%) and continuous data were described using the mean and standard deviation (mean±SD). Chi square was used to test for statistically significant difference in categorical data. *P*-values less than 0.05 were considered statistically significant.

### Data and classifications

Collected data included the following: age at first presentation, age at onset of seizures, gender, medical history, developmental history, family history, seizure semiology, physical and neurological signs, response to anticonvulsive treatment, electroencephalography recordings, neuroimaging studies, genetic testing, metabolic and any other relevant investigations.

Epileptic seizures, electro clinical syndromes and their etiologies were classified according to the scheme proposed by the ILAE in 2010.^1^ Mode of onset of seizure was considered generalized if the patient had generalized-onset seizures and generalized epileptic form discharges on EEG. Patients were classified as focal if they had focal-onset seizure. Additionally, patients with generalized tonic-clonic seizures and focal finding on neurological examination, neuroimaging or EEG, were considered to have focal seizures. Epileptic patients who had more than one seizure type were considered to have mixed epileptic seizures (**Table 1**). Patients who presented with spasms and developed later other seizure types were considered to have spasms. Patients who presented with focal or generalized seizure and later developed spasms were considered to have both spasms and other seizure types and documented in **Table 1** as mixed epileptic seizures. Patients were classified to have genetic etiology if they had strong genetic contribution such as childhood absence epilepsy or juvenile myoclonic epilepsy or if they had chromosomal disorders or genetic mutation for their epilepsy. Neurocutaneous syndromes which give rise to structural brain abnormalities and distinctive constellations were classified as having structural etiology. Moreover, focal seizures with unknown etiology were further classified according to the lobe they originate from or undetermined if not possible to refer to specific lobe which may help in clarifying this group further. Epileptic seizures were considered drug-resistant when occurred at least monthly for more than 6 months and after adequate use of 2 antiepileptic drugs.^11^ At least, one EEG was carried out on every patient; neuroimaging was carried out on 91.7% of all cases and brain MRIs were carried out on 75% of all cases (**Table 1**).

**Table 1:** Seizure types, their characteristics and main investigations of 663 Jordanian pediatric epileptic patients.

Parameters	One seizure type	Mixed seizures	All seizure types
	n (%)		
***Generalized***	248 (41.5)	103 (74.1)	351 (47.6)
Tonic Clonic	139 (23.2)	29 (20.9)	168 (22.8)
Absence	41 (6.9)	11 (7.9)	52 (7.1)
Myoclonic	24 (4.0)	28 (20.1)	52 (7.1)
Clonic	4 (0.7)	3 (2.2)	7 (0.9)
Tonic	27 (4.5)	16 (11.5)	43 (5.8)
Atonic	13 (2.2)	16 (11.5)	29 (3.9)
***Focal***	317 (53.0)	29 (20.9)	346 (46.9)
Without ICA*	59 (9.9)	9 (6.5)	68 (9.2)
With ICA	178 (29.8)	18 (12.9)	196 (26.6)
With ICA and evolving to BCS**	43 (7.2)	1 (0.7)	44 (6.0)
Without ICA* and evolving to BCS**	37 (6.2)	1 (0.7)	38 (5.2)
***Spasms***	33 (5.5)	7 (5.0)	40 (5.4)
Intractable	161/663 (24.3)		
MRI alone	389/663 (58.7)		
CT alone	111/663 (16.7)		
Both CT and MRI	108/663 (16.3)		
None	55/663 (8.3)		
***Number of patients N=663***			
One seizure type	598 (90.2)		
Mixed seizures	65 (9.8)		
***Number of seizures N=737***			
One seizure type	598 (81.1)		
Mixed seizures	139 (18.9)		

* ICA - impairment of consciousness or awareness,

** BCS - evolved to bilateral convulsive seizure

## Results

Results showed mainly mode of onset, type and etiology of epileptic seizures in Jordanian epileptic patients. A group of 663 patients were included in the study and another group of 61 patients were excluded from the study because of inadequate information. Most patients (96.5%) were evaluated in the pediatric neurology clinics after age of one year. Also, 27.1% of all epileptic patients had their first seizure before age of one year. Slight and significant increase in male to female ratio was noted (*p*<0.001) (**Table 2**).

**Table 2:** Demographic data of 663 Jordanian pediatric epileptic patients.

Demographics identifiers	N (%)
***Age groups***	
2-12 month	23 (3.5)
>1-6 years	239 (36.0)
>6-12 years	275 (41.5)
>12-18 years	126 (19.0)
***Age at seizure onset***	
<1 month	27 (4.1)
1-12 month	153 (23.0)
>1-6 year	373 (56.3)
>6-12 year	105 (15.8)
>12-18 years	5 (0.8)
***Gender***	
Male	377 (56.9)
Female	286 (43.1)
Family History	225 (33.9)

One seizure type was reported in 598 (90.2%) of all epileptic patients and the focal epileptic seizures of this group were significantly the most common 317 (53%) (*p*<0.001), followed by generalized 248 (41.5%) and epileptic spasms 33 (5.5%) (**Table 1**). Focal seizures with impairment of consciousness or awareness (F with ICA) were significantly the most common focal epileptic seizure 178/317 (56.2%) and tonic clonic seizures were the most common generalized epileptic seizures 139/248 (56%) (*p*<0.001) (**Table 1**). Infact, focal seizures are more common to present in the first year (n=73) than generalized seizures (n=58) (*p*<0.001) (**Table 3**). One third of all epileptic seizures were due to electro clinical syndromes and 60/221 (27.1%) of electro clinical syndromes were due to benign epilepsy with centro-temporal spikes (BECTS) followed by absence epilepsy 41/221 (18.6%) (**Table 4**).

**Table 3:** Age at onset of epileptic seizures, their types and aetiology.

Aetiology and type of seizures	Age of seizure onset				
	<1 m	1-12 m	>1 y	>6 y	>12-18 y	Total
***Aetiology (n=663)***						
Genetic	2	13	74	27	1	117
Structural and metabolic	20	89	134	33	2	278
Unknown	5	51	165	45	2	268
***Type of seizure (one seizure type n=598)***						
Generalized	9	49	146	41	3	248
Focal	12	61	187	56	1	317
Spasms	3	24	6	0	0	33

m - month, y - year(s)

**Table 4:** Electro clinical syndromes of 221 Jordanian pediatric epileptic patients.

Type	Aetiology			Total
	Presumed Genetic	Structural-metabolic	Unknown	
Ohtahara syndrome	0	0	1	1 (0.5)
West syndrome	2	24	7	33 (14.9)
Myoclonic epilepsy in infancy	0	0	3	3 (1.4)
Dravet syndrome	2	0	0	2 (0.9)
Myoclonic encephalopathy in non-progressive disorders	0	0	1	1 (0.5)
Panayiotopoulos syndrome	0	0	2	2 (0.9)
Epilepsy with myoclonic atonic (previously astatic) seizures	6	0	0	6 (2.7)
BECTS	0	0	60	60 (27.1)
ADNFLE	1	0	0	1 (0.5)
Late onset childhood occipital epilepsy (Gastaut type)	0	0	3	3 (1.4)
Lennox-Gastaut syndrome	1	6	1	8 (3.6)
Epileptic encephalopathy with continuous spike-and-wave during sleep	0	0	1	1 (0.5)
Landau-Kleffner syndrome	0	0	1	1 (0.5)
Childhood absence epilepsy	26	0	0	26 (11.8)
Juvenile absence epilepsy	15	0	0	15 (6.8)
Juvenile myoclonic epilepsy	12	0	0	12 (5.4)
Epilepsy with generalized tonic–clonic seizures alone	37	0	0	37 (16.7)
Progressive myoclonus epilepsies	0	0	2	2 (0.9)
Reflex epilepsies	7	0	0	7 (3.2)
**Total**	**109**	**30**	**82**	**221 (100)**

BECTS - Benign epilepsy with Centro temporal spikes, ADNFLE - Autosomal-dominant nocturnal frontal lobe epilepsy

The etiology of epilepsies are shown in **Table 5**. Apparently, structural-metabolic causes were the most common 278 (41.9%), followed by unknown causes in 268 (40.4%) and genetic causes in 117 (17.7%) patients. Genetic group causes consisted of 2 subgroups. The first included 109 electro clinical syndromes and considered to have presumed genetic causes and the second subgroup included 8 patients who had genetic disorders in which epileptic seizures do not constitute a core symptom but are rather an association to the primary phenotype. That second subgroup included the following disorders: 2 fragile x syndromes, one Prader-Willi Syndrome, one Angleman syndrome, one 46, xx-14, RING^14^ abnormality, one Rett syndrome, one Bloom syndrome and one Rubenstein-Taybi syndrome (**Table 5**).

**Table 5:** Etiology of epilepsies of 663 patients.

Etiology of epilepsies	Frequency
***Genetic n (%)***	117 (17.7)
Electro clinical syndromes (presumed genetic)	109
Others	8
***Structural – metabolic n (%)***	278 (41.9)
Perinatal insults	89
Malformation of cortical development	26
Infection	23
Neurocutaneous syndromes	23
Trauma	16
Hydrocephalus	12
Metabolic disorders	9
Vascular anomaly	7
Tumor	5
Stroke	3
***Others:***	54
Corpus collosum	10
Brain atrophy	23
Microcephaly	8
Brain cysts	10
Delay myelination	3
Distinctive constellations:	11
Mesial temporal lobe epilepsy with hippocampal sclerosis	10
Rasmussen syndrome	1.0
***Unknown n (%)***	268 (40.4)
Electro clinical syndromes	82
Other types of epilepsy:	186
Focal temporal lobe	45
Focal frontal lobe	27
Focal parietal lobe	5
Focal occipital lobe	4
Focal (undetermined origin)	19
Generalized	70
Mixed seizure	16
**Total n (%)**	**663 (100)**

Electro clinical syndromes group (n=221) was due to presumed genetic causes in 109 (49.3%) patients, followed by unknown causes 82 (37.1%) and structural-metabolic causes 30 (13.6%) (**Table 4**).

Of the structural–metabolic group, 89/278(32%) were due to perinatal disorders and were the most common causes of this group, followed by malformation of cortical development 26/278 (9.3%) (**Table 5**). The unknown causes group in our study consisted of electro clinical syndromes 82/268 (30.6%) and other types of epilepsies 186/268 (69.4%) (**Table 5**). Structural-metabolic causes significantly predominated in the first year 109/180 (60.5%) and unknown etiology predominated after the first year 217/483 (44.9%) (**Table 3**) (*p*<0.001). Moreover, **Table 3** showed that age at onset of epileptic seizures was in the first 12 years in 658/663 (99.2%) and was in the first 6 years in 553/663 (83.4%) patients.

## Discussion

There is little published research on type and etiology of epilepsy in pediatrics using the ILAE report 2010. Our study is about classification and etiology of epilepsies using the ILAE report 2010 and among few studies in the international level.^3^,^8^-^11^,^13^ In fact, 4 of these studies included pediatric epileptic patient^3^,^8^-^9^,^11^ and 2 of them were hospital-based, one of them used the 2010 ILAE report similar to our study^11^ and the other one used the previous classifications for comparison with the 2010 ILAE report.^3^ Our study is also, unique in being multi-center.

Age at onset of seizures in the first year of life in our study was 27.1%, which is less than that of Khoo’s study (42.1%),^11^ but similar to other studies.^3^,^8^,^14^ Age at onset of epilepsies in the age group >1-12 years, was documented in 72.9% of patients in our cohort, which is comparable to that reported by other studies.^8^,^11^

Most common type of epileptic seizures presenting with one seizure type was focal in our study (53%) (**Table 1**). This finding is comparable to other studies which revealed that (53.3-69%) of new onset epilepsy was focal.^11^,^14^-^15^ Also, the most common type of focal seizures was (F with ICA) (56.2%) and approximately quarter of the focal seizures evolved to bilateral convulsive seizures (BCS). Although, the ambiguity using complex partial seizures in the old classification is replaced by more meaningful terminology in the new classification of the 2010 ILAE report, the description of types of focal seizures, looks lengthy like (F with ICA evolving to BCS) and probably needs modification.^3^,^6^ Forty patients (6%) in our study had epileptic spasms and 33 of them were due to West syndrome. Other studies reported epileptic spasms in (3-8%) of patients.^8^,^11^,^16^

Electro clinical syndromes were identified in 33.3% of our epileptic patients when compared to (14-28%) in other studies.^8^,^11^,^13^ The BECTS accounted for 27.1% of all electro clinical syndrome in our study which is in contrary to what was reported by Syvertsen et al^13^ who found that absence epilepsy (both childhood and juvenile) was the most common electro clinical syndromes (36.1%) and BECTS accounted for only (18%).^13^ The high incidence of BECTS in our study may be also, attributed partially to over treatment of such entity in Jordan. Distinctive constellations were documented in (1.7%) in our study which is comparable to that reported by other studies.^8^,^11^

Structural-metabolic causes were the most common causes of epilepsies in our study (41.9%) which are comparable to other studies.^3^,^8^,^11^,^13^ Most patients of this group (87.4%) had their seizure onset in the first 6 years and 39.2% of the group had their seizures onset in the first year of life, which is slightly higher than that reported by Weirell et al^8^ (**Table 3**). Perinatal disorders were the most common causes of this group (32%), which is slightly lower than study from Saudi Arabia (40%) that was carried out long time ago in 1990,^17^ and much higher than the Norwegian study (14%).^13^ This high rate of perinatal causes of epilepsies indicates that developing countries like Jordan have to work hard on improving the perinatal care and the associated morbidity.

Unknown causes represented 40.4% of all patients in our study. Other studies reported (23.5-50%) of their patients to have unknown causes.^8^,^11^ That means there is much to be learned about epilepsy in children, because of high percentage of unknown causes hoping that the advances in genetic technology and molecular biology will decrease the size of this group.

Although genetic causes represented (17.7%) of all cases in our study (**Table 5**), some of the electro clinical syndromes included in this group of causes have little confirmed evidence of genetic basis,^18^ in the contrary, some metabolic disorders like phenyl ketoneurea and structural disorders like tuberous sclerosis are considered part of structural-metabolic group, although they have some genetic basis. Moreover, electro clinical syndromes classified under unknown like Ohtahara syndrome and BECTS have possible genetic contribution.^19^-^20^

Drug-resistant epilepsies were documented in 24.3% of our patients which is more than reported by Rasmos-Lizana et al (17%)^21^ and less than khoo study (45%)^11^ and Boonluksiri et al(42%).^22^

Despite the clarity of terminology in ILAE 2010 report, when compared to old classification, it was difficult for the project team to use the lengthy terminology for focal seizures. Obviously, the 2010 classification gave better insight to the understanding of the type of epileptic seizures, etiology of epilepsies, particularly, genetic causes which are significantly growing field, where greater integration of genetics into clinical decision making is an emerging trend^23^ and distinctive constellations as possible surgical candidates.^24^

### Limitation of the study

Potential weakness of our study was that the classification and etiology were performed mainly by one pediatric neurologist, though the whole data were reviewed by the first author. As mentioned in the study design, whenever necessary, Patients and/or their medical records were reviewed again, by the first author. Also, we have not been able to find many hospital-based studies using the revised terminology proposed by the ILAE in 2010 to compare their results with ours. However; we considered the new categories of genetic, structural-metabolic and unknown in 2010 report correspond to the previous idiopathic, symptomatic, and cryptogenic terminology.^13^ Moreover, the nature of our hospital-based study cannot give optimal epidemiological data. Obviously, in resource–limited countries like Jordan, some modification in classification of ILAE 2010 report is required due to limitation in availability of advanced genetic investigations.^10^-^11^

In conclusion, our study as the first study from the Middle East using the ILAE 2010 report and unique in being hospital-based and multicenter on the international level, demonstrated that focal epilepsies were the most common type of pediatric epilepsy, the structural-metabolic causes were the most common causes of epilepsy, the genetic causes were mainly due to presumed genetic causes. Larger population-based studies using the current and future classifications and terminology of the ILAE for epileptic seizures may give more optimal epidemiological data about types and etiologies of epilepsies.
